# Gene expression profile of androgen modulated genes in the murine fetal developing lung

**DOI:** 10.1186/1477-7827-8-2

**Published:** 2010-01-08

**Authors:** Eva Bresson, Tommy Seaborn, Mélissa Côté, Geneviève Cormier, Pierre R Provost, Bruno Piedboeuf, Yves Tremblay

**Affiliations:** 1 Laboratory of Ontogeny and Reproduction, CHUQ, CHUL, Laval University, Quebec City, Quebec, Canada; 2 Department of Obstetrics and Gynaecology, Faculty of Medicine, Laval University, Quebec City, Quebec, Canada; 3 Department of Pediatrics, Faculty of Medicine, Laval University, Quebec City, Quebec, Canada; 4 Centre de Recherche en Biologie de la Reproduction (CRBR), Laval University, Quebec City, Quebec, Canada; 5 INSERM U413/EA4310, Laboratory of Cellular and Molecular Neuroendocrinology, European Institute for Peptide Research (IFRMP), International Associated Laboratory Samuel de Champlain, University of Rouen, France

## Abstract

**Background:**

Accumulating evidences suggest that sex affects lung development. Indeed, a higher incidence of respiratory distress syndrome is observed in male compared to female preterm neonates at comparable developmental stage and experimental studies demonstrated an androgen-related delay in male lung maturation. However, the precise mechanisms underlying these deleterious effects of androgens in lung maturation are only partially understood.

**Methods:**

To build up a better understanding of the effect of androgens on lung development, we analyzed by microarrays the expression of genes showing a sexual difference and those modulated by androgens. Lungs of murine fetuses resulting from a timely mating window of 1 hour were studied at gestational day 17 (GD17) and GD18, corresponding to the period of surge of surfactant production. Using injections of the antiandrogen flutamide to pregnant mice, we hunted for genes in fetal lungs which are transcriptionally modulated by androgens.

**Results:**

Results revealed that 1844 genes were expressed with a sexual difference at GD17 and 833 at GD18. Many genes were significantly modulated by flutamide: 1597 at GD17 and 1775 at GD18. Datasets were analyzed by using in silico tools for reconstruction of cellular pathways. Between GD17 and GD18, male lungs showed an intensive transcriptional activity of proliferative pathways along with the onset of lung differentiation. Among the genes showing a sex difference or an antiandrogen modulation of their expression, we specifically identified androgen receptor interacting genes, surfactant related genes in particularly those involved in the pathway leading to phospholipid synthesis, and several genes of lung development regulator pathways. Among these latter, some genes related to Shh, FGF, TGF-beta, BMP, and Wnt signaling are modulated by sex and/or antiandrogen treatment.

**Conclusion:**

Our results show clearly that there is a real delay in lung maturation between male and female in this period, the latter pursuing already lung maturation while the proper is not yet fully engaged in the differentiation processes at GD17. In addition, this study provides a list of genes which are under the control of androgens within the lung at the moment of surge of surfactant production in murine fetal lung.

## Background

Accumulating evidence suggests that sex affects lung development and physiology. Indeed, sex hormones appear to exert regulatory effects on human lung development and maturation during both fetal and neonatal periods. During the fetal period, male lung maturation is delayed compared with female and surfactant production appears earlier in female than in male fetal lungs [1]. In preterm infants, surfactant deficiency greatly contributes to the development of respiratory distress syndrome (RDS) [1] and its resulting morbidity such as bronchopulmonary dysplasia (BPD), patent ductus arteriosus, and long-term neurological disabilities [2,3]. Consequently, male neonates have an increased risk of developing RDS and a higher risk of morbidity and mortality due to RDS compared with female neonates [4]. Moreover, a higher incidence in male was also observed for the development of new BPD, a pulmonary pathology of the neonate for which RDS is not always an anterior event [5].

Furthermore, substantial experimental data support a role for sex hormones in lung development regulation. Indeed, several genes have been shown to be expressed with a sexual dimorphism within murine fetal maturing lung [6]. More precisely, androgens have been shown to exert inhibitory effects on lung maturation both *in vitro *[7] and *in vivo *[8,9]. For instance, maternal treatment with the androgen dihydrotestosterone (DHT) during fetal rabbit development inhibits surfactant phospholipids production in the female fetal lung, while maternal treatment with the antiandrogen flutamide increases surfactant phospholipids production in the male fetal lung [9]. In human, androgens delay alveolar epithelial type II (PTII) cell maturation in males compared with females [10]. Similarly, androgens delay fetal lung surfactant production in a variety of species [11] by a mechanism involving transforming growth factor-β (TGF-β) signalling pathway [12].

To build up a better understanding of the biomolecular mechanisms underlying androgens effect on lung development, we investigated the expression of genes showing a sexual dimorphism and those modulated by the presence of androgens at the moment of surge of surfactant production in murine fetal lung. For achieving this goal, microarrays were performed on fetal mouse lungs of both sexes harvested at gestational day 17 (GD17) and GD18 (term is GD19), preceded by maternal daily injections of the pure antiandrogen flutamide or the vehicule solution (control) from GD10. In the mouse, GD17 and GD18 represent the transition between canalicular and saccular stages of lung development, which overlaps the surge of surfactant production. Then, the present investigation has been designed to provide valuable insights in the study of the signalling mechanisms leading to surfactant synthesis and pulmonary maturation. In general, the results show that thousands of genes are transcriptionally modulated within the developing lung in response to the fetal androgenic status. More specifically, we report the modulation of androgen receptor interacting genes, surfactant related genes and particularly those involved in the pathway leading to phospholipid synthesis, and several genes of lung development regulator pathways.

## Methods

### Animals and housing

Protocols were approved by the Animal Care and Use Committee and the Institutional Review Board of the Centre de Recherche du Centre Hospitalier Universitaire de Québec (protocol no. 2005-156). BALB/c mice (*Mus musculus*) aged from 63 to 70 days and certified pathogen free were purchased from Charles River Laboratories (St-Constant, QC, Canada). These were housed in a room maintained at 22 +/- 3°C, 50 +/- 20% relative humidity and on a 12-hours cycle (07:15-19:15 hours) of fluorescent lighting (300 Lux). Commercial diet (Global 18% Protein Rodent Diet, Teklad, Montréal, QC, Canada) and tap water were provided *ad libitum*. New animals were acclimatized to these conditions for 7 to 14 days prior to be timely mated in a one hour mating window as previously described [13].

### Experimental procedures

Flutamide antiandrogen (kindly provided by Dr Fernand Labrie) was dissolved in a saline vehicule solution (0.9% NaCl) containing 1% gelatin (W/V) (ACP Chemicals, Saint-Léonard, QC, Canada) and 10% dimethylsulfoxide (DMSO) (Sigma, St. Louis, MO). Pregnant females received a daily sub-cutaneous injection of 200 μl of flutamide (1 mg) or vehicule solution from GD10 to the day prior to harvesting day. Pregnant females were sacrificed by exposure to CO_2 _at GD17 or GD18. From each fetus, lungs and a rear leg were harvested and rapidly frozen on dry ice and then stored at -80°C until use.

### Fetal sex determination

Fetal sex was identified by examination of the genital tract with a dissecting microscope at 15× magnification and confirmed by PCR amplification of the male-specific *Sry *gene [14] (GenBank: X67204) from fetal rear leg. DNA was purified by phenol-chloroform extraction followed by ethanol precipitation. A hot start procedure with Taq DNA Polymerase kit (Roche Diagnostics, Laval, QC, Canada) was achieved and PCR reactions were performed according to the protocol of the manufacturer with 0.04 nM of each *Sry *primer (forward: nucleotide position 36-55, 5'-TATGGTGTGGTCCCGTGGTG-3'; reverse: nucleotide position 337-317, 5'-ATGTGATGGCATGTGGGTTCC-3'), resulting in a 282 nucleotides amplicon. The following PCR conditions were used: 94°C for 5 min. and 72°C for 10 min. followed by 34 cycles of 94°C for 1 min., 65°C for 1 min. and 72°C for 1 min. Final extension was done at 72°C for 10 min. Agarose gel electrophoresis was used for amplicon visualization.

### RNA extraction and sampling

Total RNA was extracted from fetal lung using Tri-reagent, a mixture of phenol and guanidine thiocyanate in a monophasic solution (Molecular Research Center, Cincinnati, OH) as described previously [15]. Each RNA sample was purified on a CsCl gradient as described [16], using a TLA 120.2 rotor in an Optima MAX centrifuge (Beckman, Mississauga, ON, Canada). The quality of RNA was monitored by micro-capillary electrophoresis (Bioanalyser 2100, Agilent Technologies, Mississauga, ON, Canada). RNA samples from three fetuses of the same sex but from distinct litters were then pooled (n = 1). Each experimental group was triplicated (n = 3). The 6 experimental groups, resulting in 18 RNA pools for microarray hybridization, were the following: vehicule-injected males harvested at GD17 (17 m) or GD18 (18 m), vehicule-injected females harvested at GD17 (17 f) or GD18 (18 f), and flutamide-treated males harvested at GD17 (17 flut) or GD18 (18 flut).

### Preparation of probes

For each RNA pool, 20 μg of total RNA was converted to cDNA by using SuperScript II reverse transcriptase (Invitrogen), and T7-oligo-d(T)24 (Geneset) as a primer. T7 BioArray High Yield RNA Transcript Labeling Kit was used to produce biotinylated cRNA. The mixture (20 μl final volume) was incubated at 37°C for 5 h with gentle mixing every 30 min. Labelled cRNA was purified using a RNeasy Mini Kit (Qiagen) according to the protocol of the manufacturer. Purified cRNA was fragmented into segments of 20-300 nucleotide length by incubation in a fragmentation buffer (100 mM potassium acetate, 30 mM magnesium acetate, 40 mM Tris-acetate pH 8.1) for 20 min at 94°C. The quality of cRNA amplification and cRNA fragmentation was monitored by micro-capillary electrophoresis (Bioanalyser 2100, Agilent Technologies, Mississauga, ON, Canada).

### Microarray hybridization, scanning, and analysis

Fifteen micrograms of fragmented cRNA was hybridized for 16 h at 45°C with constant rotation, using a mouse oligonucleotide array MOE430 2.0 (Genechip, Affymetrix, Santa Clara, CA). After hybridization, chips were processed by using the Affymetrix GeneChip Fluidic Station 450 (protocol EukGE-WS2v5_450). Staining was made with streptavidin-conjugated phycoerythrin (SAPE; Molecular Probes), followed by amplification with a biotinylated anti-streptavidin antibody (Vector Laboratories), and by a second round of SAPE. Chips were scanned using a GeneChip Scanner 3000 G7 (Affymetrix) enabled for High-Resolution Scanning. Images were extracted with the GeneChip Operating Software (Affymetrix GCOS v1.4). Quality control of microarray chips was performed using the AffyQCReport software [17]. A comparable quality between microarrays was demanded for all microarrays within each experiment.

The MOE430 2.0 microarray provides coverage of over 45,000 probe sets corresponding to about 39,000 transcripts and variants. The probe sets were selected from sequences derived from GenBank, dbEST and RefSeq. The sequence clusters were created from the UniGene database (Build 107, June 2002) and then refined by analysis and comparison with the publicly available draft assembly of the mouse genome from the Whitehead Institute Center for Genome Research (MSCG, April 2002). Data sets have been deposited in GEO (GSE18135). 

### Statistical analysis

The background subtraction and normalization of probe set intensities was performed using the Robust Multiarray Analysis (RMA) method described by Irizarry et al. [18]. To identify differentially expressed genes, gene expression intensities were compared using a moderated *t*-test and a Bayes smoothing approach developed for a low number of replicates [19]. To correct for the effect of multiple testing, the false discovery rate was estimated from *p *values derived from the moderated *t*-test statistics [20]. The analysis was performed using the affylmGUI Graphical User Interface for the limma microarray package [21].

## Results

**Figure 1:**
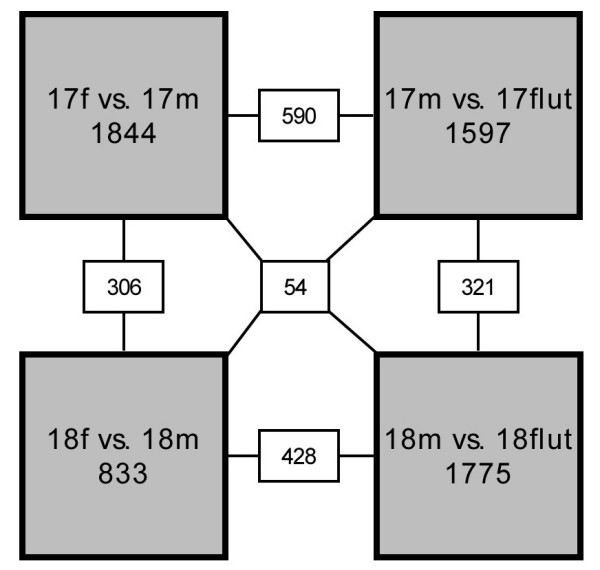
**Overview of microarray results obtained from fetal mouse lungs**. Gene expression levels were compared at both GD17 and GD18 in males versus females (17 f *vs*. 17 m and 18 f *vs*. 18 m) as well as in males versus flutamide-treated males (17 m *vs*. 17 flut and 18 m *vs*. 18 flut). The number of genes differentially expressed for each comparison appears in grey boxes. White boxes contain the number of differentially expressed genes shared by comparisons to which they are connected. Flut: flutamide treated males, m: males, f: females.

**Figure 2:**
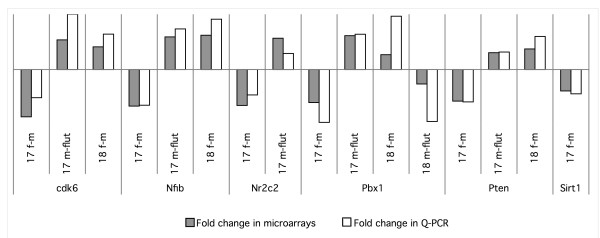
**Microarray validation by quantitative real-time PCR (Q-PCR)**. Q-PCR were achieved for six genes randomly selected among those for which microarrays reveal a sexual difference and/or flutamide sensitivity: cyclin-dependant kinase 6 (Cdk6), nuclear factor I/B (Nfib), pre B-cell leukemia transcription factor 1 (Pbx1), phosphatase and tensin homolog (Pten), sirtuin 1 (Sirt1), and nuclear receptor subfamily 2, group C, member 2 (Nr2c2). Gene expression data are shown as a relative fold-change of the second experimental group over the first experimental group in each comparison. Genes showing increased expression in male (m) compared to female (f) or in male (m) compared to flutamide treated male (flut) are placed above the axis and those with decreased expression below the axis.

Fetal lung gene expression was compared at both GD17 and GD18 between males and females (17 f *vs*. 17 m and 18 f *vs*. 18 m) and between males and flutamide-treated males (17 m *vs*. 17 flut and 18 m *vs*. 18 flut) (Fig. 1). Analysis indicates that more than twice of the genes were expressed with a sexual difference at GD17 than at GD18 (1844 *vs*. 833) while flutamide modulated the expression level of a comparable number of genes at GD17 and GD18 (1597 and 1775, respectively). There was a substantial overlap between the genes expressed with sexual dimorphism and those affected by flutamide (590 at GD17 and 428 at GD18). In addition, 306 genes showed sexual difference at both GD17 and GD18 and 321 genes were modulated by flutamide at both studied gestational ages. Finally, 54 genes showed expression modulation in all the four comparison protocols. In order to assess the validity of the microarrays, we achieved real-time quantitative PCR for a few genes randomly selected among those presenting sexual difference and/or flutamide sensitivity by using cDNA from the same experimental groups than those used for microarray experiments. The genes considered for quantitative PCR were cyclin-dependant kinase 6 (Cdk6), nuclear factor I/B (Nfib), pre B-cell leukemia transcription factor 1 (Pbx1), phosphatase and tensin homolog (Pten), sirtuin 1 (Sirt1), and nuclear receptor subfamily 2, group C, member 2 (Nr2c2). In each case, the trend observed by quantitative PCR confirmed the trend observed in microarray results (Fig. 2).

**Figure 3:**
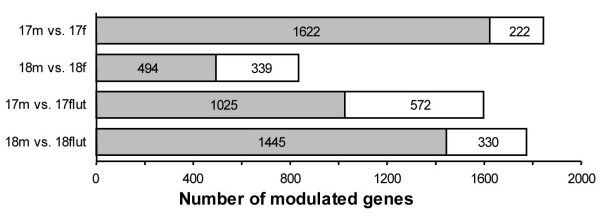
**Modulation of fetal pulmonary gene expression by sex and antiandrogen treatment**. Grey boxes contain the number of genes highly expressed in male fetal lungs harvested at GD17 or GD18 (17 m and 18 m) when compared with timely corresponding female fetal lungs (17 f and 18 f) or flutamide-treated male fetal lungs (17 flut and 18 flut). Numbers within white boxes represent the number of genes for which the expression is lower in male fetal lungs than in the compared group. Flut: flutamide treated males, m: males, f: females.

At GD17, 88% of the genes expressed with a sexual difference were more highly expressed in male lungs than in female lungs and this proportion decreased to 59% at GD18 (Fig. 3). Among the 1597 genes significantly modulated at transcriptional level by flutamide at GD17, 36% were up-regulated and 64% were down-regulated. This tendency is amplified at GD18 where only 19% of the modulated genes were transcriptionally up-regulated by flutamide while 81% were down-regulated.

**Figure 4:**
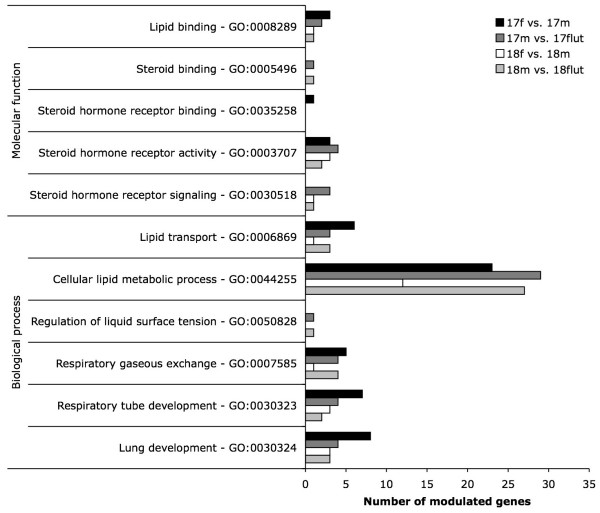
**Profile of biological processes and molecular functions of genes transcriptionally modulated by sex and antiandrogen treatment**. Modulated genes were analyzed on Gene Ontology website www.geneontology.org and results were reported for genes included within categories (GO:category number) related to surfactant production and regulation, pulmonary development and physiology, lipid processing, and androgen receptor signalling. Flut: flutamide treated males, m: males, f: females.

Genes showing expression modulation by sex and/or antiandrogen treatment were analyzed by using the Gene Ontology website http://www.geneontology.org in order to delineate to which biological processes and/or molecular functions they are associated. In accordance with the scope of this study, focus was placed on genes related to surfactant production and regulation, pulmonary development and physiology, lipid processing, and androgen receptor (AR) signalling (Fig. 4). Surprisingly, a relatively low number of genes related to these processes showed sexual difference and/or flutamide sensitivity, except for cellular lipid metabolic process. Indeed, 23 and 12 genes related to this biological process were expressed with a sex difference at GD17 and GD18, respectively, and 29 and 27 genes were affected by flutamide treatment at these gestational ages.

The subset of genes presenting sex difference and being affected by flutamide at the same gestational age (590 at GD17, 428 at GD18) were regrouped in accordance with their pattern of relative pulmonary expression level among experimental groups (Table 1). For the majority of those genes, the expression level was higher in males than in females (571 at GD17 and 344 at GD18; summation of genes with expression patterns #2 and #5). However, at GD18 the proportion of genes highly expressed in females represents about 20% (84 genes; expression patterns #3 and #4) of the total genes whereas at GD17 it counts for only 3% (19 genes; expression patterns #3 and #4). Some of those genes are related to lipid processing, hormones, and lung function. Since they represent potential important players in the action of androgens in fetal lung development, they are specifically presented in Table 2.

**Table 1:** Expression patterns of genes transcriptionally modulated by sex and antiandrogen treatment within fetal lung

Expression pattern	Relative expression level among groups^1^	Number of genes	
		GD17^2^	GD18
#1	flut > m > f	0	0
#2	m > flut > f	359	93
#3	flut > f > m	7	21
#4	f > flut > m	12	63
#5	m > f > flut	212	251
#6	f > m > flut	0	0

^1^Groups are flutamide treated males (flut), males (m), and females (f)

^2^GD = Gestational day

**Table 2:** Genes related to hormones, lipid processing, and lung function that are modulated by sex and antiandrogen treatment

**Biological function**	**Gene symbol**	**Gene name**	**Expression pattern^1^**
**GESTATIONAL DAY 17**			
Steroid	Hsd17b12	Hydroxysteroid (17-beta) dehydrogenase 12	#2 (m > flut > f)
Androgen	Ext1	Exostoses (multiple) 1	#2 (m > flut > f)
Glucocorticoid	Glcci1	Glucocorticoid induced transcript 1	#5 (m > f > flut)
Lipid binding	Prkca	Protein kinase c, alpha	#5 (m > f > flut)
Lipid binding	Ncam1	Neural cell adhesion molecule 1	#5 (m > f > flut)
Lipid binding	Gpc6	Glypican 6	#2 (m > flut > f)
Steroid hormone receptor activity	Nr6a1	Nuclear receptor subfamily 6, group a, member 1	#2 (m > flut > f)
Steroid hormone receptor activity	Nr2c2	Nuclear receptor subfamily 2, group c, member 2	#2 (m > flut > f)
Cellular lipid metabolic process	Sgpp1	Sphingosine-1-phosphate phosphatase 1	#2 (m > flut > f)
Cellular lipid metabolic process	Pbx1	Pre b-cell leukemia transcription factor 1	#5 (m > f > flut)
Cellular lipid metabolic process	Hsd17b12	Hydroxysteroid (17-beta) dehydrogenase 12	#2 (m > flut > f)
Cellular lipid metabolic process	Hadhb	Hydroxyacyl-coenzyme A dehydrogenase/3-ketoacyl-coenzyme A thiolase/enoyl-coenzyme A hydratase (trifunctional protein), beta subunit	#2 (m > flut > f)
Cellular lipid metabolic process	Ggtla1	Gamma-glutamyltransferase-like activity 1	#3 (flut > f > m)
Respiratory gaseous exchange	Pbx3	Pre b-cell leukemia transcription factor 3	#2 (m > flut > f)
Respiratory tube development	Nfib	Nuclear factor i/b	#2 (m > flut > f)
Respiratory tube development	Foxp1	Forkhead box P1	#5 (m > f > flut)
Embryonic development	Kif1b	Kinesin family member 1b	#2 (m > flut > f)
Embryonic development	Ext1	Exostoses (multiple) 1	#2 (m > flut > f)
**GESTATIONAL DAY 18**			
Lipid binding	Rock1	Rho-associated coiled-coil forming kinase 1	#5 (m > f > flut)
Lipid binding	Nr5a2	Nuclear receptor subfamily 5, group a, member 2	#5 (m > f > flut)
Steroid hormone receptor activity	Nr5a2	Nuclear receptor subfamily 5, group a, member 2	#5 (m > f > flut)
Cellular lipid metabolic process	Pip5k3	Phosphatidylinositol-3-phosphate/phosphatidylinositol 5-kinase, type III	#4 (f > flut > m)
Cellular lipid metabolic process	Pbx1	Pre b-cell leukemia transcription factor 1	#4 (f > flut > m)
Cellular lipid metabolic process	Nr5a2	Nuclear receptor subfamily 5, group a, member 2	#5 (m > f > flut)
Cellular lipid metabolic process	Idi1	Isopentenyl-diphosphate delta isomerase	#2 (m > flut > f)
Cellular lipid metabolic process	Elovl6	Elovl family member 6, elongation of long chain fatty acids (yeast)	#2 (m > flut > f)
Cellular lipid metabolic process	Dhcr24	24-dehydrocholesterol reductase	#2 (m > flut > f)
Cellular lipid metabolic process	Crls1	Cardiolipin synthase 1	#5 (m > f > flut)
Lipid transport	Osbpl9	Oxysterol binding protein-like 9	#4 (f > flut > m)
Respiratory gaseous exchange	Fut8	Fucosyltransferase 8	#4 (f > flut > m)
Respiratory tube development	Gli1	Gli-kruppel family member gli1	#2 (m > flut > f)

^1^Genes are situated among their corresponding expression patterns presented in Table 1. Flut: flutamide treated males. m: males. f: females.

**Figure 5:**
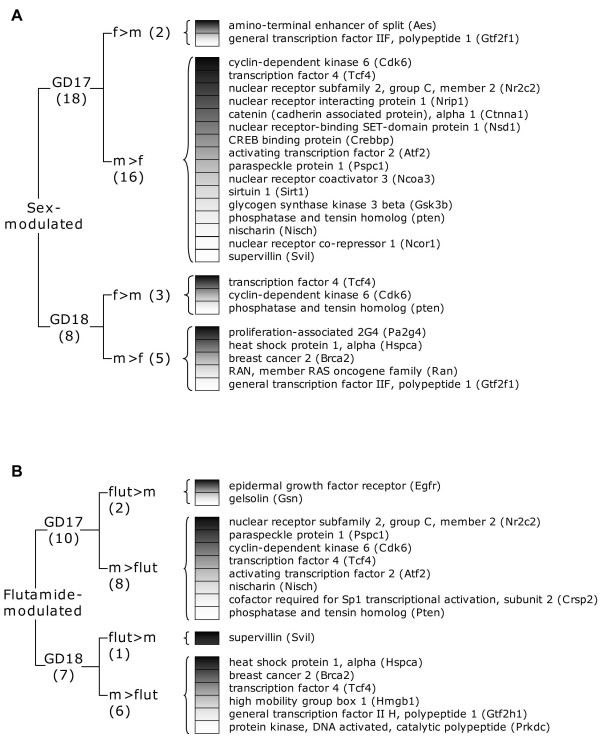
**Genes associated to androgen receptor signalling that are modulated by sex or antiandrogen treatment**. Genes for which the pulmonary expression level is modulated by sex (A) or antiandrogen treatment (B) at GD17 or GD18. The color gradient within boxes represents the relative magnitude in the differential expression level between compared groups; darker is the box, greater is the difference. Flut: flutamide treated males, m: males, f: females.

In addition to the genes presenting both sex- and flutamide-driven modulations, some others presented either a sex difference or a transcriptional modulation by flutamide treatment at GD17 or GD18. Many of these genes are related to androgen receptor signalling and are more specifically known as co-regulators in the formation of AR complex (Fig. 5). Among these genes, an important proportion was modulated at GD17, the majority of them being more highly expressed in males than in females (Fig. 5A) or than in flutamide-treated males (Fig. 5B). Conversely, only 5 genes were more highly expressed in females than in males (2 at GD17 and 3 at GD18) and only 3 genes were up-regulated within the fetal male lung by flutamide treatment (2 at GD17 and 1 at GD18).

Several other genes presenting either a sex difference or transcriptional modulation by flutamide treatment at GD17 or GD18 are known to be involved in pulmonary development signalling pathways or in surfactant synthesis or regulation (Table 3). Indeed, our results show that some components of signalling pathways mediating lung development and/or maturation present a transient sex difference in expression, being more highly expressed in males at GD17 but not at GD18. Genes related to surfactant synthesis and regulation also showed a sex regulation that seems to be more important at GD17 that at GD18. More specifically, this sex regulation at GD17 favors high expression level for females for genes involved in sphingolipid synthesis while genes involved in phosphatidyl choline synthesis or in IGF signalling tend to be more highly expressed in male than in female lungs. Among the surfactant-related genes, surfactant associated proteins A, B, and C were transcriptionally up-regulated by flutamide treatment at GD17, reaching a level comparable to the one of female lungs at GD18.

**Table 3:** Genes associated with pulmonary development or surfactant that are modulated by sex and/or antiandrogen treatment

**Process^1^**	**Symbol**	**Gene name**	**Modulation^2^**
**LUNG DEVELOPMENT REGULATORS**			
Shh signaling	Gli1	GLI-Kruppel family member GLI1	17 m>17 f; 18 m>18 f
Shh signaling	Gli3	GLI-Kruppel family member GLI3	17 m>17 f; 18 m>18 f
FGF signaling	Fgf10	Fibroblast growth factor 10	17 m>17 f
TGF-β Signaling	Tgfbr3	Transforming growth factor, beta re ceptor III	17 m>17 f
TGF-β Signaling	Tsc22d1	TSC22 domain family, member 1	17 m>17 f
TGF-β Signaling	Tgfb2	Transforming growth factor, beta 2	17 flut>17 m
BMP signaling	Bmpr2	Bone morphogenic protein receptor, type II	17 m>17 f; 18 m>18 flut
BMP signaling	Gdf10	Growth differentiation factor 10	17 m>17 flut
BMP signaling	Gdf15	Growth differentiation factor 15	17 flut>17 m
Wnt signaling	Gsk3b	Glycogen synthase kinase 3 beta	17 m>17 f
Wnt signaling	Ctnna1	Catenin (cadherin associated protein), alpha 1	17 m>17 f
Wnt signaling	Tcf4	Transcription factor 4	17 m>17 f
Wnt signaling	Crebbp	CREB binding protein	17 m>17 f
**SURFACTANT RELATED GENES**			
Phosphatidyl cholin synthesis	Lpin2	Lipin 2	17 m>17 f; 18 m>18 f
Phosphatidyl cholin synthesis	Chka	Choline kinase alpha	17 m>17 f; 17 m>17 flut; 18 m>18 f; 18 m>18 flut
Phosphatidyl cholin synthesis	Cds2	CDP-diacylglycerol synthase (phosphatidate cytidylyltransferase) 2	18 flut>18 m
Phosphatidyl cholin synthesis	Aytl2 (Lpcat1)	Lysophosphatidylcholine acyltransferase 1	17 flut>17 m
Sphingolipids synthesis	Mgll	Monoglyceride lipase	17 f>17 m
Sphingolipids synthesis	Aldh9a1	Aldehyde dehydrogenase 9, subfamily A1	17 f>17 m
Sphingolipids synthesis	Pnliprp2	Pancreatic lipase-related protein 2	17 f>17 m
Sphingolipids synthesis	Sgpp1	Sphingosine-1-phosphate phosphatase 1	17 f>17 m
Sphingolipids synthesis	Dusp11	Dual specificity phosphatase 11 (RNA/RNP complex 1-interacting)	17 f>17 m
Sphingolipids synthesis	Crls1	Cardiolipin synthase 1	17 f>17 m; 18 m>18 f
Sphingolipids synthesis	Gba	Glucosidase, beta, acid	17 f>17 m
Surfactant proteins	Sftpa1	Surfactant associated protein A1	17 flut>17 m; 17 flut>17 f; 18 m>17 m; 18 f>17 f
Surfactant proteins	Sftpb	Surfactant associated protein B	17 flut>17 f; 18 m>17 m; 18 f>17 f
Surfactant proteins	Sftpc	Surfactant associated protein C	17 flut>17 f; 18 m>17 m; 18 f>17 f; 18 m>18 flut
Surfactant proteins	Sftpd	Surfactant associated protein D	18 m>17 m; 18 f>17 f
**SURFACTANT REGULATION**			
Miscellaneous regulators	Klf5	Kruppel-like factor 5	17 flut>17 m; 17 flut>17 f
Miscellaneous regulators	Ltbp1	Latent transforming growth factor beta binding protein 1	17 m>17 f
Miscellaneous regulators	Pten	Phosphatase and tensin homolog	17 m>17 f; 17 m>17 flut
Miscellaneous regulators	Magi1	Membrane associated guanylate kinase, WW and PDZ domain containing 1	17 m>17 f
Miscellaneous regulators	Ptges2	Prostaglandin E synthase 2	17 f>17 m
IGF signaling	Igfbp2	Insulin-like growth factor binding protein 2	17 m>17 flut
IGF signaling	Igfbp5	Insulin-like growth factor binding protein 5	17 m>17 flut
IGF signaling	Igf1	Insulin-like growth factor 1	17 m>17 flut
IGF signaling	Igfr2 (Fcgr2b)	Fc receptor, IgG, low affinity IIb	17 m>17 flut

^1^Shh: sonic hedgehog, FGF: fibroblast growth factor, TGF-β: transforming growth factor beta, BMP: bone morphogenetic protein, Wnt: wingless integration site, PC: phosphatidyl choline, IGF: insulin growth factor.

^2^Flut: flutamide treated males, m: males, f: females.

## Discussion

We previously achieved a microarray analysis of the sex difference in fetal mouse lungs at GD15.5, GD16.5, and GD17.5 by using overnight mating [6]. This protocol revealed only 83 genes presenting a sex difference at one or more of the studied time points. The much more larger number of genes showing a sex difference we report here could be explained by the mating window which was reduced to 1 h for the current study. The mouse gestational period is very short (term GD19). Therefore, because the sex difference results from a delay in modulation, the gestation period corresponding to this delay should be very short. As a consequence, overnight mating window previously used may have restrained the amount of genes showing a sex difference. Moreover, in the current study, we also performed microarray analysis of male fetal lungs following flutamide or vehicle (negative control) administration in order to identify genes in the male fetal lung that were directly or indirectly under the control of androgens.

### Androgen receptor interacting genes

The AR, which mediates androgen effects, is a nuclear transcription factor that binds to androgen-responsive elements as well as to co-activators and general transcription factors to control transcription of androgen-regulated genes [22]. As a ligand-dependent transcription factor, the AR is activated by binding to one androgen molecule: testosterone or DHT. AR is present in both male and female lungs [23]. Moreover, in the developing lung, there is active androgen metabolism where androgen synthesis and inactivation take place [23,24] making the lung a candidate organ for direct androgen control. The lack of a functional AR in tissues of the testicular feminized mice (Tfm) is well-documented and no difference was observed in the phosphatidyl choline/sphingomyelin ratio between Tfm males and normal females [8]. In Tfm males, this ratio was not altered by exogenous androgen while it is lowered in the female. This finding strongly indicates that androgen acts to alter fetal lung development via the AR. The formation of AR transcriptional complex requires its functional and structural interaction with several co-regulators [25]. Our results show that, during the period overlapping the surge of surfactant synthesis, there is an obvious difference in AR signalling regulation between male and female lungs. Indeed, the higher androgen concentration occurring in male lungs is accompanied by a notably higher expression level of many co-factors/co-regulators of AR. Moreover, many of them are androgen-regulated, as testify their flutamide-sensitive expression. One of those cofactors, Pten, is particularly interesting while it is able to regulate AR signalling in both direct and indirect manner. Pten interacts directly with AR to suppress androgen-induced AR nuclear translocation and it also regulates AR activity via a PI3K/Akt-dependent pathway [26]. In Drosophila, PTEN was demonstrated to suppress cell growth and G1/S progression by down-regulating the PI3K/Akt pathway and to inhibit the G2/M transition through an alternative mechanism, perhaps involving regulation of the cytoarchitecture [27]. Thus, Pten appears to be an important regulator in the timing of cell proliferation and maturation.

### Lung development regulators

During embryonic and pseudoglandular stages of lung development, sonic hedgehog (Shh) plays an important positive role. Indeed, lack of Shh signalling leads to severe pulmonary hypoplasia [28]. The output of hedgehog signalling is mediated by the modulation of the Gli transcriptional activators and their repressors. The mammalian Gli gene family consists of three members, Gli1, Gli2, and Gli3 [29], which are expressed in the lung mesenchyme during the pseudoglandular stage of development [30]. While Gli1 and Gli2 are transcriptional activators of Shh signalling, Gli3 is a bipotential transcription factor, and the repressor form of Gli3, generated as a result of proteolytic cleavage, is activated in the absence of Shh signalling [31]. As a consequence, we do not know whether the higher expression of Gli3 observed for males in our study tend to increase Shh activation. In contrast, the fact that Gli1expression is also higher in males clearly suggests that Shh signalling should be higher in male than in female developing lungs.

Some of the signalling components of pulmonary epithelial-mesenchymal interaction, essential for branching morphogenesis, are also more expressed in male than in female lungs, such as Fgf10 and Bmpr2 genes. Members of the fibroblast growth factor (FGF) family and in particular Fgf10 are potent chemotactic signalling molecules. Fgf10 elicits lung budding and branching morphogenesis [32]. Disruption of Fgf10 results in a complete lack of lung parenchyma distal to the trachea [33] and, *in vitro*, Fgf10 alone is both necessary and sufficient for morphogenesis in mesenchyme-free endodermal explants [32]. Fgf10 shows a sex-dependent expression at GD17 with higher levels in males, suggesting that the FGF10-dependent growth is still continuing in the male lung at this time of gestation. In contrast to FGFs, the role of TGF-β is thought to be inhibitory for embryonic lung branching morphogenesis. Tgfbr3, a TGF-β receptor participating in TGF-β-mediated negative regulation of lung organogenesis [34], is strongly expressed in male lung at GD17. Another TGF-β-dependent protein involved in differentiation process is Tsc-22. This protein enhances TGF-β-dependent erythroid cell differentiation [35] and modulates Smad activity, which may influence cell differentiation in many different tissues, including the lung. Like Tgfbr3, Tsc-22 is more highly expressed in male lungs on GD17. One of TGF-β isoforms, TGF-β2 (coded by mouse Tgfb2 gene) is transcriptionally induced by flutamide treatment at GD17. Lungs of the Tgfb2 null mice revealed no gross morphological defects prenatally, but display collapsed conducting airways postnatally [36], so Tgfb2 seems to be essential for the development and maintenance of the respiratory function.

Contrary to Fgf10 and Gli which are expressed in the mesenchyme, Bmp proteins (bone morphogenetic proteins) are expressed in the adjacent pulmonary epithelium. Bmps constitute the largest group of cytokines belonging to the TGF-β superfamily. Originally, they were identified as molecules regulating growth and differentiation of bone and cartilage. However, they also control growth, differentiation, and apoptosis in a diverse number of cell lines, including mesenchymal and epithelial cells, regulating embryogenesis and contributing to the maintenance and repair of adult tissues [37]. Bmp proteins signal through two subtypes of receptors: Bmpr1 and Bmpr2. Bmpr2 is distinguished from other TGF-β superfamily members in that it initiates intracellular signalling in response to the specific ligands Bmp-2, Bmp-4, Bmp-6, Bmp-7, growth and differentiation factor 5 (Gdf-5), and Gdf-6 [38]. We report that Bmpr2 gene is highly expressed in male lungs compared to female lungs at GD17, and compared to flutamide-treated males at GD18. None of the mentioned Bmp or Gdf ligands is regulated according to sex, but Gdf10 and Gdf15 show androgen-responsive expression, being respectively down- and up-regulated by flutamide treatment at GD17. Gdf15 is an important determinant of collecting duct lengthening in mouse, and evidences suggest that it is also involved in development [39]. It was also shown to be regulated during keratinocyte differentiation [40] while Gdf15 mRNA and protein expression patterns correlate with proliferative activity and cellular differentiation during the various stages of normal prostate development [41]. Gdf10 was localized to areas of programmed cell death in the limb where it mediates retinoic receptor signalling [42]. This gene has also putative tumor suppressor functions, being hypermethylated and down-regulated in lung cancers [43]. Therefore, these two Gdf genes seem to be involved in the promotion (Gdf15) or the inhibition (Gdf10) of proliferation and differentiation in fetal lung development.

Other signalling proteins that regulate cell-cell interactions in many embryonic tissues belong to the Wnt (wingless-related) family. Wnts signal through multiple pathways, the most well-characterized being the canonical β-catenin/TCF pathway [44]. In this pathway, secreted Wnt proteins bind to Frizzled receptors on cell membranes, activating Disheveled protein, which in turn inactivates Gsk3. Giving that Gsk3 phosphorylates β-catenin leading to the subsequent degradation of this molecule, its inactivation inhibits phosphorylation of β-catenin. Hypophosphorylation stabilizes β-catenin, which is then transported to the nucleus where it heterodimerizes with members of the TCF family of transcription factors and Creb binding protein (Crebbp) to activate downstream target genes. β-catenin, a central molecule of canonical Wnt signalling, has been shown to localize in the undifferentiated primordial epithelium, differentiating alveolar epithelium, and adjacent mesenchyme [45]. In mouse, β-catenin dependent signalling is essential to the formation of the peripheral airways of the lungs. According to our results, genes encoding for Gsk3, β-catenin, Tcf4, and Crebbp are all more highly expressed in male at GD17, suggesting involvement of these genes in some differentiating processes presenting a temporal delay for one sex.

### Surfactant related genes

Five decades ago, it was already suggested that surfactant deficiency could cause hyaline membrane disease, currently called RDS. This disease of prematurely born infants is due to a lack of surfactant [46]. The surfactant complex is composed by lipids (90%) and proteins (10%) [47]. Among the surfactant lipids, the most abundant (70%) is phosphatidylcholine (PC), principally as dipalmitoylphosphatidylcholine (DPPC). Among the enzymes involved in PC synthesis, we report that lipin 2 (Lpin2) and choline kinase α (Chka) are more highly expressed in male than female lungs. In addition, Chka is more highly expressed in male than in flutamide-treated male. The Cds2 gene encoding for the enzyme responsible for CDP-diacylglycerol synthesis does not show a sex difference in expression, while it is up-regulated by flutamide at GD18. The main gene responsible for DPPC synthesis, Aytl2, is up-regulated by flutamide at GD17. Thus, the main pathway leading to PC and DPPC synthesis seems to be regulated by an androgen-sensitive mechanism. A pathway susceptible to be more active in female than in male lungs on GD17 is sphingolipids synthesis for which genes Mgll, Aldh9a1, Pnliprp2, Sgpp1, Dusp11, Crls1, and Gba are all more strongly expressed in female than in male lungs.

The surfactant proteins play crucial roles in the structure, function, and metabolism of surfactant. Four proteins enter in the composition of surfactant: Sftpa1, Sftpb, Sftpc, and Sftpd (also known as SP-A, SP-B, SP-C, and SP-D, respectively). According to our microarray results, the expression of the four surfactant protein genes increases over time in both male and female lungs, but does not show any significant sexual difference. Compared to male lungs, Sftpa1 expression was augmented in flutamide-treated males at GD17, while Sftpc mRNA levels were higher in males than in flutamide-treated males at GD18. Thus, our results cannot totally exclude the participation of androgens in the control of expression of these genes.

Some factors involved in lung morphogenesis are also involved in surfactant synthesis regulation. Kruppel-like factor 5 (Klf5) gene is involved in lamellar body formation, in the stability of DPPC and Sftpb levels in late gestation in mouse, and in lung maturation during the saccular stage of development [48]. Klf5 is regulated by androgens since its expression is higher in flutamide-treated male than in male and female at GD17. Klf5 influences paracrine signalling between lung epithelium and mesenchyme, including TGF-β pathway. TGF-β was shown to inhibit expression of Sftpa1, Sftpb, and Sftpc surfactant proteins [49]. TGF-β is produced as a latent complex, which must be activated by cleavage [50]. The latent transforming growth factor-β-binding protein (Ltbp1) has been shown to facilitate secretion of latent TGF-β [51] and to target latent TGF-β to the extracellular matrix for storage [52]. Ltbp1 protein cleavage may provide a mechanism for release of the latent TGF-β from ECM [53]. The fact that Ltbp1 is more highly expressed in males suggests that TGF-β pathway activation could be more pronounced in males compared to females. As alleged by others, TGF-β-induced inhibition of endodermal morphogenesis is associated with inhibition of cell proliferation, which is in large part due to increased expression of Pten [54]. Control males expressed Pten at higher levels than females and flutamide-treated males at GD17. However, at GD18, flutamide caused an increase in Pten expression in males, suggesting a switch in the Pten expression peak from male to female at GD18. Pten binds the scaffolding protein Magi-1 [55] which shows a sexual difference with a male prevalence at GD17. Magi-1/2/3 proteins are implicated in recruiting Pten to intercellular junctions [56,57] and may play a crucial role in organizing thymoma viral proto-oncogene (AKT) and phosphatidylinositol 3-kinase (PI3K) signalling complexes that control cell growth, differentiation, and dissemination [55,58].

Several members of insulin-like growth factor (IGF) family show flutamide-responsive expression. Indeed, genes coding for Igf binding proteins Igfbp2 and Igfbp5, as well as Igf1 and Igf receptor 2 (Igfr2) are more highly expressed in males when compared to flutamide-treated males. Among them, only the Igfr2 gene exhibits a sexual difference, being more highly expressed in males than in females. Another factor involved in surfactant regulation is prostaglangin E2 (Ptges2), which increases surfactant secretion in rat [59]. In our study, Ptges2 is more highly expressed in females at GD17, suggesting that surfactant secretion could be up-regulated as a consequence of Ptges2 expression.

## Conclusion

While early lung development is characterized by cell proliferation, late lung development is predominantly governed by cell differentiation processes during which proliferation is markedly reduced. The fact that male-GD17 lungs showed increased expression of some proliferative signals, like Fgf10, compared to female lungs put forward the thesis that male lungs are not yet fully engaged in the differentiation processes at GD17, but Tgfbr3 receptor expression suggests a tight control of cell proliferation. Even if TGF-β is a negative regulator of airway branching morphogenesis in early lung development, its signalling is active in several tissue types in the lung during normal late development [60]. We also have shown that four genes related to the Igf pathway are more highly expressed in male lungs at GD17. This pathway is involved in the mechanism of lung maturation leading to surfactant synthesis. Thus, male lungs at GD17 appear to continue their growth and to start their maturation at the same time in preparation to birth. The male-increased expression of components from two opposite signals, one promoting cell proliferation and another one promoting cell differentiation, suggests a progressive evolution from the proliferative to differentiation states in some cell types within male lungs at GD17.

Furthermore, many genes involved in lipid processing have been shown to be expressed with sexual dimorphism. Some genes involved in this process have a sexual difference at GD17 or GD18, and some show flutamide-sensitive modulation. Even though it is difficult to confirm that the phospholipids synthesis is delayed for one sex, it is well known that the male disadvantage in lung maturation is mediated by androgens and that these steroids lead to a reduction in choline incorporation into DPPC in vitro [7,61], which is in line with our results showing that the enzyme responsible for DPPC production is up-regulated by antiandrogen, thus confirming a negative effect of androgens on this process. It should be mentioned that the sex difference in surfactant lipid levels may also be attributed in part to pulmonary expression of some apolipoproteins [62].

Generally, the male lung seems more "transcriptionally active" than the female lung at GD17. Indeed, the majority of genes differentially expressed between sexes are more highly expressed in males. This transcriptional activity difference suggests that some processes are delayed in male lungs compared to females. Accordingly, there is an obvious decrease in transcriptional activity in male lungs between GD17 and GD18 while, during the same period, the number of genes showing a higher expression level in female lungs remain stable.

Taken together, our results are compatible with a delay in expression of pathways related to late development for one sex, with female lungs already more advanced in their maturation process, and male lungs showing an important transcriptional activity for pathways related to proliferation. By identifying several genes that are modulated according to sex and/or by antiandrogen in male fetal lungs, this study provides a significant number of candidate genes under the control of androgens that are likely to be involved in the delay in lung maturation observed in males. Further investigations of these candidates will be helpful in the understanding of this sexual dimorphism and have the potential to give valuable insights relevant to the prevention and treatment of short- and long-term consequences resulting from a wide range of pathologies associated with lung immaturity, such as RDS and BPD.

## Competing interests

The authors declare that they have no competing interests.

## Authors' contributions

EB carried out the microarray data analysis and drafted the manuscript. TS carried out mice matings, injection protocol, tissue harvesting, sample preparation for microarray experiments and Q-PCR, and also participated to design study and to draft the manuscript. MC participated in mice matings and injection protocol. GC carried out Q-PCR confirmations. PRP participated in the conception and design of the study. BP participated in the conception and design of the study. YT conceived and coordinated the study. All authors read and approved the final manuscript.
